# Social Exclusion Among Official Language Minority Older Adults: A Rapid Review of the Literature in Canada, Finland and Wales

**DOI:** 10.1007/s10823-021-09433-z

**Published:** 2021-06-08

**Authors:** Fredrica Nyqvist, Emilia Häkkinen, Alexandre Renaud, Louise Bouchard, Cynog Prys

**Affiliations:** 1 Faculty of Education and Welfare Studies, Social Policy, Åbo Akademi University, Strandgatan 2, 65100 Vasa, Finland; 2 Faculty of Social Sciences, University of Ottawa, Ottawa, Canada; 3 School of History, Philosophy and Social Science, Bangor University, Bangor, Wales

**Keywords:** Canada, Finland, Older adults, Rapid review, Social exclusion, Wales

## Abstract

It has been suggested that older adults from minority linguistic and ethnic communities face higher risks of being socially excluded. The aim of this review was, therefore, to explore and review social exclusion studies conducted among official language minority older adults in three countries, namely Canada, Finland and Wales. A rapid review approach was used to review scientific literature in line with six social exclusion domains. The literature searches were made in Finnish, Swedish, English, French and Welsh and were restricted to research published within the timeline of 2001 – September 2019 and yielded 42 articles. The included studies were categorized into three different domains: socioeconomic influences, social participation and societal conditions. Converging and diverging patterns of social exclusion in old age were identified between the linguistic minorities. Linguistic barriers regarding access to health care and receiving health information were common across the three linguistic contexts, whereas exclusion from social participation was noticed amongst the linguistic minorities in Canada and Wales. Some connections between belonging to a linguistic minority and being exposed to a lower socioeconomic status and higher poverty risk were made, however, these findings were not robust across all three countries. The findings indicated that experiences of exclusion could be considered fairly common among linguistic minority older adults. We conclude that the research evidence presented in the review sheds light on issues of social inequality in old age between linguistic majorities and minorities, thus identifying important aspects of social exclusion to guide future research as well as policy and practice.

## Introduction

Social exclusion is a major concept in the social policy discourse and research (Millar, 2007; Wilson, 2006). For example, the European Union and its member states have agreed to combat poverty, discrimination and social exclusion in order to allow equal service access and opportunities for all citizens (European Parliament, 2020). Further, although social exclusion is often used to denote exclusion from labor market participation (e.g. Gómez-Torres et al., 2019), the definition of social exclusion is both comprehensive and multidimensional and is embedded in the economic, political as well as cultural and social structures of society (Walker & Walker, 1997), which involves a recognition of micro- (individual agency), meso- (community) and macro-level processes (society). Moreover, the social exclusion research literature is extensive. Previous scientific reviews on social exclusion have examined conceptualizations and definitions (Millar, 2007; Rawal, 2008), its relation to negative health outcomes (Morgan et al., 2007; Wright & Stickley, 2013), its conceptualization, distribution and prevalence across various geographical contexts (Grant et al., 2000; Philip & Shucksmith, 2003) and demographic groups including younger (Harden et al., 2006) and older people (Van Regenmortel et al., 2016; Walsh et al., 2017).

In general, older people face a higher risk of being socially excluded due to age-related changes, such as an increasing risk of functional limitations, health issues, and loss of a partner, family and friends. Moreover, ageism or age discrimination is common in various societies and is seen as one key barrier to older people’s inclusion (Stuckelberger et al., 2012). Previous studies conducted in different contextual settings suggest that older people from minority ethnic communities are especially vulnerable to being socially excluded due to a higher risk of poverty and marginalisation (e.g. Heikkinen, 2011; McCann et al., 2013; Victor et al., 2012). Although exclusion from social and health care services from a linguistic perspective has been acknowledged (e.g. Martin et al., 2018), the social exclusion of older official linguistic minority groups still seems to play a subordinate role in the social gerontology literature (Walsh et al., 2017). A review of existing social exclusion literature amongst older official linguistic minorities will, therefore, make a fruitful contribution to this line of research and add to the understanding of the complexity and plurality of the ageing experience.

### An overview of Canada, Finland and Wales

For the purpose of this review three countries with more than one official or national language, namely Canada, Finland and Wales, were studied. The legal framework for linguistic rights differs between the countries as well as the general conditions of the linguistic minority and some of the features of the selected countries will be described in more detail below.

Canada, a federation of 10 provinces and three territories, has a population of 38 million people and recognizes English (75.4%) and French (22.8%) as official languages (Canadian Heritage, 2019; Statistic Canada, 2021). Consequently, all federal institutions are required to provide services in English and French upon request (Official Languages Act, 1988). At the provincial level, French is the official language of Quebec, the only Francophone majority province in the country, and of New-Brunswick, which is officially bilingual. Other provinces have varying requirements regarding the use and availability of French language services depending on regional demographics. With regards to official language minority communities (OLMCs), there are approximately 1 million Anglophones living in Quebec and there is roughly the same number of Francophones dispersed throughout the remainder of Canada, but with a significant concentration in the province of Ontario and New Brunswick (Canadian Heritage, 2019). Different legal principles have guided the development of Canadian language legislation, such as the constitutional principle of the protection of minorities, the restoration of linguistic rights, and the principle of substantive equality as well as sociological concepts of institutional completeness, cultural autonomy, and ethnolinguistic vitality (Chouinard, 2016).

Finland is a bilingual country that constitutionally recognizes Swedish and Finnish as the national languages (The Constitution of Finland, 1999). According to the Language Act of 2003 that was passed to ensure the “right of everyone to use his or her own language, either Finnish or Swedish, before courts of law and other authorities, and to receive official documents in that language” (The Constitution of Finland, 1999 §17), public authorities must respond to the cultural and social needs of both linguistic groups on equal grounds. Approximately 290 000 (5%) of a total of 5.5 million are Swedish-speaking, whereas 88% of the population is Finnish-speaking. Other linguistic minorities account for 7% of the population. In addition, Finnish municipalities are either officially unilingual or bilingual, depending on the size of the official language minority community. Of the total number of 311 municipalities in Finland in 2018, 33 were classified as bilingual (15 with Swedish as the majority language and 18 with Finnish as majority language) and 16 were classified as unilingual Swedish. The duties of regional and state authorities to provide services in both languages depend on the linguistic status of the municipality as unilingual or bilingual (Language Act, 2003 5§). In bilingual municipalities, authorities are obliged to offer services in both languages.

In Wales, the Welsh language is spoken by 562,000 people, which represents 19% of the population (Office of National Statistics, 2012). As a result, Welsh is often considered a minority language in Wales. However, the Welsh language was granted official status in Wales as a part of the Welsh Language Measure of 2011 (Welsh Government, 2011). Both the British Government and the Welsh Government have enacted legislation to enable the use of Welsh within public services in Wales. The Welsh Language Act (1993) legislated that the Welsh language was to be treated on a basis of equality with English within public services. This was further enhanced by the Welsh Language Measure (2011) which stated that Welsh was to be treated no less favourably than English within the public sector. While the majority of the Welsh population speak English as their first language, in some parts of the country Welsh is considered as the first language and English the second. While the communities in the rural Northern and Western parts of Wales contain the highest percentage of Welsh speakers, such as in the counties of Gwynedd (65%) and the Isle of Anglesey (57%), the more urban communities in the South East also contain high numbers (in absolute terms) of Welsh speakers (Office of National Statistics, 2012). Nonetheless, census data points to a decrease in the communities with a higher concentration of Welsh speakers (ibid) raising concerns for the future viability of Welsh. As a result, the Welsh Government published their ambitious strategy to double the number of Welsh speakers by 2050 (Welsh Government, 2017). This represents the political will, and the broad public support, for the maintenance and revitalisation of the Welsh language in Wales.

It is evident that the included countries have unique linguistic climates and that the linguistic conditions vary significantly between them. Although they do not allow for linear comparisons, the three countries still clearly make interesting linguistic contexts for a review of social exclusion, the results of which could be used to explore issues of inequality within and between various linguistic groups.

As mentioned, even if social exclusion is a multidimensional concept that encompasses social, economic, political and cultural domains and that lacks a strong definition (Littlewood et al., 1999; Millar, 2007; Rawal, 2008), various authors emphasize the importance to consider the concept as relative, meaning that the exclusion of any defined group should be assessed in relation to another group. Further, they agree that social exclusion involves interacting processes so that older people might be excluded by the actions of other individuals or institutions. Social exclusion is also dynamic and processual with individuals experiencing exclusion at different times or in different situations. Thus, social exclusion is not narrowly defined as material deprivation, marginalisation or poverty, but also includes exclusion from participation in social and civic life. Some have proposed that participation and/or social connectedness are the opposite of exclusion rather than inclusion whereas some have argued that inclusion and exclusion are inseparable sides of the same coin (Rawal, 2008; Taket et al., 2009). According to a recent review (Walsh et al., 2017), social exclusion literature amongst older people considers six thematic social exclusion domains: social relations (e.g. social networks and support, social opportunities); material and financial resources (e.g. poverty, income, pensions); service, amenities and mobility (e.g. health and social care services, general services, housing); civic participation (e.g. volunteering, political participation, citizenship); neighbourhood and community (e.g. service, amenities, social and relational aspects) and socio-cultural aspects (e.g. identity exclusion, ageism and symbolic exclusion). It is common that exclusion in one domain also affects other domains (Walsh et al., 2017), so that for example the exclusion of material and financial resources might limit the possibilities to be civically engaged.

To summarize, our review offers valuable knowledge synthesis and insight into the social exclusion literature from a language perspective by reviewing studies conducted in three different multilingual contexts. Using a rapid review methodology, the current review builds on the results of Walsh et al. (2017) by exploring research along the six conceptual domains. By assessing scientific social exclusion literature amongst older linguistic minorities, the results can guide future research by highlighting possible gaps and needs, and by serving as a framework for guiding policy and practice when it comes to actions seeking to reduce social exclusion.

The aim of the rapid review was, therefore, to explore and review studies with a focus on social exclusion among official language minority older adults in Canada, Finland and Wales in order to gain insight into how social exclusion has been approached and addressed in previous research.

## Methods

### Identification of Studies

A rapid research methodology of published scientific literature was undertaken to explore current knowledge on social exclusion amongst older linguistic minorities living in Canada, Finland and Wales. Rapid reviews have been described as a knowledge synthesis method in which components of a systematic review process are simplified and omitted at times (Ganann et al., 2010; Khangura et al., 2012). They can be seen as streamlined approach to systematic review methods and yield valid information on the research topic addressed, however, with less certainty than a full systematic review, making it valuable but less rigorous. Further, rapid reviews can be conducted within a shorter timeframe compared to a systematic review; while the time span for conducting a systematic review could be a year, rapid reviews can be conducted within weeks or months (Khangura et al., 2012). The two methodological approaches additionally separate regarding the synthesis of the findings. While systematic reviews can be characterized by a qualitative summary of the findings and occasionally an additional meta-analysis, rapid reviews are identified by a briefer, descriptive categorization and summary of the data (Khangura et al., 2012). Thus, rapid reviews have been identified as a research method that produces timely, user friendly and trustworthy evidence that allows for a comprehensive literature exploration (Ganann et al., 2010; Khangura et al., 2012). The rationale for conducting a rapid review in this study was to provide an overview of the literature targeting the broad concept of social exclusion within a relatively short timeframe by simplifying the search methods.The search process was initiated in September 2019 and concluded in March 2020. The searches were initially made in *Swedish, Finnish* and *English* in September 2019. Complementary searches in *French* and *Welsh* to identify Canadian and Welsh publications were made in March 2020. It was conducted in the following electronic databases: Social Science Citation Index, Academic Search Premier, Social Science Premium Collection and Google Scholar.

Search terms (with Boolean logic) used in the search process are presented in Table 1. The search strategy was developed with the help of an Academic librarian and was restricted to scientific publications with abstracts in Finnish, Swedish, English, French and Welsh that had been published within the timeline of January 2001– September 2019. However, concerning publications in Welsh, no studies addressing the specific subject of the review were detected.

**Table 1:** Keywords employed in the literature search

Word group 1	Word group 2	Word group 3	Word group 4
Canada Finland Wales	Minorities Minority groups Language Linguistics Language contact Linguistic minorities Bilingualism Multilingualism Sociolinguistics	Social exclusion Social isolation Social deprivation Inequalities Relative deprivation	Older people Old age Middle age Ageing Aging Elderly people Retirees Senior citizens Elder*

**Notes**: Words within groups combined with OR

Groups combined with AND

Keywords translated to Finnish, Swedish, French and Welsh

**Limits: **Published within the timeline of January 2001 – September 2019

Abstracts in Finnish, Swedish, English, French and Welsh

Ages 60 +

### Inclusion and Exclusion Criteria

Irrespective of methodology (i.e. quantitative or qualitative), studies were included if they targeted older adults aged 65 years and older and if they assessed any of the defined domains of social exclusion (Walsh et al., 2017) that targeted official linguistic minorities (such as the Francophone minority living outside Quebec/Canada, the Anglophone minority living in Quebec/Canada, the Swedish speaking minority in Finland, and the Welsh-speakers in Wales). However, studies involving younger age groups alongside adults aged 65 years and older were also included if the studies had a sufficient focus on older adults. Since we limited our search to scientific peer-reviewed articles, studies from the grey literature and book chapters were excluded.

### Search Outcome

Titles and abstracts were scanned by three researchers (EH, KB, AR) each responsible for a specific country, to make initial assessments of relevance based on inclusion and exclusion criteria. If inclusion of the studies was unclear; a final decision was made in the consultation with another researcher (FN). The title and abstract based searches resulted in 48 studies in the initial search phase in September 2019 and 40 additional studies in the complementary phase conducted in March 2020. After concluding the initial and complementary search phase, the 88 selected studies were reviewed by two researchers (EH, AR). At this point, further exclusions were made on the basis of the exclusion criteria, resulting in a total of 42 studies to be included in the review. The characteristics and findings of these studies were summarized, including the languages they sought to study, sample size, age range, study purpose, type of social exclusion/inclusion presented, methodology, study results and conclusions.

## Results

The themes for this rapid review were initially planned to follow the categorization presented by Walsh et al. (2017), in which the concept of social exclusion is divided into six categories. However, such categorization of the studies was deemed unfeasible for this review, as the included studies would not directly fall into these specific categories. As a result, we generated three broader domains: *socioeconomic influences, social participation and societal conditions*. Socioeconomic influences domain encompassed studies focusing on material and financial resources whereas the studies focusing on social relations, civic participation, neighborhood and community were grouped into the social participation theme. Finally, the societal conditions domain included studies assessing services, amenities and mobility and socio-cultural aspects.

Of the 42 studies included, seven focused on the socioeconomic domain of social exclusion, whereas 20 assessed the participation domain and 28 focused on the societal domain. Twenty-five of the studies were conducted in Canada, 11 in Finland and six in Wales. One of the studies used a cross-country comparative approach and included both Finland and Canada (Holmqvist & van Vaerenbergh, 2013). In the case of Wales, no studies with a specific socioeconomic focus were detected. Concerning Finland and Wales, a majority of the studies were categorized into the participation domain of social exclusion (mainly studies on social capital, participation and sense of community), whereas for Canada the most prevalent domain was the societal one (service use and access). A number of studies were categorized in several domains of social exclusion, as was the case when studies focused on all three domains or for example on both the participation and societal domains (e.g. Dupuis-Blanchard et al., 2015; Martin et al., 2018).

The majority of the included studies targeted older adults specifically, while some focused on a broader age range by also including a younger population. The studies included in the review appeared to be quite evenly divided between those employing a multiple language perspective (studies including comparisons between the minority and majority) and those using a single language perspective (studies focusing on the minority only). The multiple language focus was more prevalent among the Finnish studies, while in the case of Wales and Canada, a single language focus was more common. A significantly higher number of the included studies was based on quantitative rather than qualitative data. In total, 25 were quantitative whereas 11 were qualitative; moreover, six studies were based on a mix of quantitative and qualitative methodology. The categorization of studies is presented in further detail in Table 2.

**Table 2:** Description of the studies included in the review

Country	Author	Study target group		Language perspective		Social exclusion domains			Methodology		
		Older adults	Mixed	Multiple	Single	Socio- economic	Participation	Societal	Qualitative	Quantitative	Mixed
Canada	Alimezelli et al. 2014	X		X			X	X		X	
	Armstrong et al. 2015	X		X				X		X	
	Batista et al. 2019	X		X				X		X	
	Bouchard et al. 2012	X			X			X	X		
	Bouchard et al. 2015	X		X		X				X	
	Cairney & Krause 2005	X		X		X		X		X	
	De Moissac et al. 2015		X		X			X		X	
	Dupuis-Blanchard & Villalon 2013	X		X				X	X		
	Dupuis-Blanchard et al. 2014	X			X		X	X			X
	Dupuis-Blanchard et al. 2015	X			X	X	X	X	X		
	Forgues et al. 2011	X			X			X		X	
	Gosselin & Viens 2006	X			X		X		X		
	Guerin et al. 2018	X		X		X		X		X	
	Kennedy & McIsaac 2008	X			X			X	X		
	Michaud et al. 2015	X		X			X	X			X
	Pakzad et al. 2012	X			X			X		X	
	Pakzad et al. 2013	X		X				X		X	
	Savard & Marchand, 2016	X			X			X	X		
	Savard et al. 2018		X		X			X		X	
	Simard et al. 2015	X			X			X	X		
	Simard 2019	X			X			X	X		
	Stout et al. 2008		X	X				X		X	
	Stout et al. 2009		X	X		X		X		X	
	Van Kemenade et al. 2015	X		X		X		X		X	
	Zunzunegui et al. 2004	X			X		X			X	
Finland	Eriksson-Backa, 2008	X			X			X		X	
	Holmqvist & van Vaerenbergh 2013		X	X				X		X	
	Härtull & Nygård 2014	X		X		X				X	
	Kulla et al. 2006	X			X		X	X	X		
	Nilsson et al. 2015	X		X			X			X	
	Nyqvist & Nygård 2012	X		X			X			X	
	Nyqvist et al. 2012	X		X			X			X	
	Nyqvist et al. 2013a; Nyqvist et al. 2013b	X		X			X			X	
	Nyqvist et al. 2013; Nyqvist et al. 2013	X		X			X			X	
	Nyqvist & Nygård 2016	X		X			X			X	
	Nyqvist et al. 2016		X	X			X			X	
Wales	Burholt & Naylor 2005	X		X			X	X			X
	Charles & Aull Davies 2005		X		X		X				X
	Day et al. 2010		X		X		X	X			X
	Hughes et al. 2009		X		X		X	X			X
	Martin et al. 2018	X			X		X	X	X		
	Winter & Burholt 2018	X			X		X		X		

A descriptive synthesis of the domain results – socioeconomic influences, social participation and societal conditions – is presented next.

### Socioeconomic Influences

Overall, research focusing on socioeconomic exclusion among minority older adults was limited, with only seven studies targeting this domain. Regardless, correlations between socioeconomic status and belonging to a linguistic minority were detected in the single Finnish study and the six Canadian studies categorized into the socioeconomic domain. For example, there was a moderate amount of evidence suggesting connections between linguistic minority status and higher rates of objective poverty (income based), lower education/employment rates, disparities in socioeconomic status and higher odds of living in rural areas (e.g. Bouchard et al., 2015; Härtull & Nygård, 2014; van Kemenade et al., 2015). Such socioeconomic factors have further been reported to constitute barriers for aging in place, to affect the possibilities to receive high-quality care and to be linked to higher rates of psychological distress (Cairney & Krause, 2005; Dupuis-Blanchard et al., 2015; Guerin et al., 2018).

In terms of experiences of poverty, research evidence from the studies conducted in Canada made connections between belonging to the official language minority group and having a lower socioeconomic status, lower education/employment rates and a higher risk for inadequate income (e.g. Bouchard et al., 2015; Guerin et al., 2018). The Canadian studies categorized into this domain largely focused on the experiences of Francophone minorities rather than Anglophone minorities. Associations between knowledge of the majority language and possibilities for higher education and employment were reported (Bouchard et al., 2015; Dupuis-Blanchard et al., 2015). A higher income and educational level were further considered to be among the essential factors that enable staying independent in old age and thus contribute to better chances for aging in place (Dupuis-Blanchard et al., 2015). Lower income, limitations in educational attainment and higher odds of rural living among linguistic minority Francophone older adults may be linked to more frequent use of long-term care rather than home care (Guerin et al., 2018).

Additionally, research evidence from a Finnish study implied that older adults belonging to the Swedish speaking linguistic minority were more likely to report somewhat higher rates of objective poverty (income based), whereas subjective poverty (experienced) was reported to be slightly more common among the Finnish speaking majority (Härtull & Nygård, 2014). Both experiences of objective and subjective poverty were reported to be more common among older women in linguistic minority and majority groups respectively as compared to older men (Härtull & Nygård, 2014). Women have generally been reported to face higher risks for socioeconomic exclusion both among the Swedish speaking minority in Finland and the Francophone minorities in Canada (Härtull & Nygård, 2014; van Kemenade et al., 2015).

### Social Participation

The greatest variations and differences in the findings across the three linguistic contexts were detected in the social participation domain. Some central aspects were relevant for the three multilingual countries; however, contextual differences as well as country-specific issues and characteristics were also highlighted in the studies falling into this social exclusion domain. The most frequently occurring concepts across all three countries were social capital, social networks, social position, community cohesion, active aging and self-rated health (e.g. Alimezelli et al., 2013; Nilsson et al., 2015; Nyqvist & Nygård, 2016; Winter & Burholt, 2018).

Several authors have examined social participation in relation to health within the linguistic minority context of Canada (Alimezelli et al., 2013; Dupuis-Blanchard et al., 2014; Gosselin & Viens, 2006; Stout et al. 2008; Stout et al., 2009; Zunzunegui, 2004). While linguistic minority status was not independently correlated with self-rated health (SRH) for older adults, variables such as the sense of belonging to the community as well as the vitality and concentration of the community affected the experiences of SRH (Alimezelli et al. 2013; Dupuis-Blanchard et al. 2014). However, some of these variables affected minority Francophone and Anglophone older adults differently; for example, the concentration of the minority was negatively associated to SRH among Francophones (presumably due to the lack of specialized health services in some areas), but not among Anglophones (Alimezelli et al. 2013). While one study did not find statistically significant differences between genders (Alimezelli et al. 2013), another observed that certain social determinants influenced perceived health for minority Francophone men and women differently (Dupuis-Blanchard et al. 2014). Further, social interaction could also exert an impact on health (Gosselin & Viens, 2006; Zunzunegui et al., 2004). In the Eastern Townships in Quebec, minority Anglophone older adults may have engaged in their communities through volunteerism, allowing them to maintain social networks and trust, i.e. social capital, which further provided them with a sense of well-being (Gosselin & Viens, 2006). The absence of social activities has been associated with lower ratings of SRH among older adults whether living in a minority context or not (Zunzunegui 2004).

A number of studies conducted in Canada focused on the perspective of nursing home residents. Given that the majority of Canadian nursing homes outside of Quebec operate in English, the older Francophone population may be more prone to social exclusion. Group cohesion among French-speaking residents did not seem to be prioritized, as a sample of predominantly Anglophone nursing homes in Ontario, New-Brunswick and Nova-Scotia were reported to tend to group individuals together based on their health status rather than having sections reserved for linguistic groups (Michaud et al. 2015). While some staff members and residents alike indicated that services could be provided in either official language due to the fact that most residents are bilingual, certain Anglophone homes indicated that activities sometimes did take place in French, that French-speaking employees could provide services in the official minority language, and that meals could be adapted to the Francophone or Acadian culture (Michaud et al. 2015). Additionally, the proximity of the nursing home to older adults’ family members was the most cited decision factor for Francophones when choosing a home (Michaud et al. 2015). In turn, housing options, access to a variety of services and social support were variables that contributed to allowing French-speaking minority older adults to age in place (Dupuis-Blanchard et al. 2015).

Hyyppä and Mäki (2001, 2003) conducted studies in the early 2000s on social capital in relation to health in the Swedish- and Finnish-speaking general adult population. They assessed social capital by measures of trust, social networks and associational activities. Their results showed that the minority seemed to possess higher rates of social capital and tighter social networks compared with the Finnish speaking population. In addition, social capital explained part of the health advantages observed amongst the Swedish speakers. Their initial work inspired social capital studies also amongst older people and several of the included studies in the review have assessed social capital and social networks in later life (e.g. Kulla et al. 2006; Nyqvist et al. 2016). These studies have confirmed higher levels of social capital amongst Swedish-speaking older adults and show that social capital seems to have beneficial health features (e.g. Nyqvist & Nygård, 2016; Nyqvist et al. 2012). Strengthening such health resources has been reported to play an essential part in implementing health-promotive nursing strategies for linguistic minority older adults (Kulla et al. 2006). The substantial amount of social capital the minority possesses as well as their tight-knit social networks further contributed to higher rates of leisure engagement, association activities and mastery among Swedish-speaking older adults (e.g. Nilsson et al. 2015; Nyqvist & Nygård, 2016; Nyqvist, Forsman, et al., 2013; Nyqvist, Nygård, et al., 2013). Arguably, national policies and legislations have so far been rather successful in providing opportunities for active ageing also for the Swedish-speaking minority.

Concerning the Welsh studies, sense of community and community cohesion were the most frequently emerging themes in the social participation domain (Burholt & Naylor, 2005; Charles & Aull Davies, 2005; Day et al. 2010; Winter & Burholt, 2018). High levels of Welsh communication, low levels of immigration and geographical mobility, close-knit kinship networks and local organizations have all been reported to contribute to the sense of community and foster feelings of citizenship among Welsh-speaking older adults (Burholt & Naylor, 2005; Charles & Aull Davies, 2005). Experiences of cultural exclusion among older Welsh adults has been reported to stem from conflicts between cultural identity and prevailing norms, values and behaviours, which could further cause a decline in sense of belonging, safety, life satisfaction and community cohesion (Winter & Burholt, 2018). The use of the Welsh language has been reported to play a central part in the Welsh identity and community cohesion, and Welsh-speaking older adults have reported that the ability to use one’s own language feels more comfortable and “homely”, for example, in service situations (Burholt & Naylor, 2005; Hughes et al. 2009). Connections between linguistic barriers and social isolation were made in previous research (Martin et al. 2018). According to a study investigating linguistically and culturally congruent care for Welsh-speaking older adults with dementia, the absence of linguistic congruity in the care environment might have led to social isolation, which further increased the risk for depression, decreasing quality of life and hampered self-confidence (Martin et al. 2018). One of the Welsh studies focused on the integration of English-speaking minorities into Welsh communities. Even though a majority of the English-speaking incomers (i.e. England-born individuals who have migrated to Wales). reported a somewhat effortless adjustment to the Welsh community, some of the English-speakers expressed feelings of alienation and isolation (Day et al. 2010). It has been reported that a certain distance was maintained between the two language groups, in which the Welsh language played a central part (Day et al. 2010). Distinctions and divisions between the two groupings were experienced to be rooted in cultural differences, lack of success in acquiring skills in the Welsh language and a distance that was being maintained to participating in Welsh politics (Day et al. 2010).

### Societal Conditions

The largest number of the included studies – especially concerning Canada – fell under the societal conditions domain of social exclusion. The main findings in this domain were relatively similar across all three linguistic contexts explored in this study. Obstacles to service access in one’s native language as well as linguistic barriers while using both high- and low-involvement services were frequently reported (e.g. Alimezelli et al. 2013; Bouchard et al. 2012; Eriksson-Backa, 2008; Holmqvist & van Vaerenbergh, 2013; Hughes et al. 2009). Moreover, several studies assessed differences in service quality, differences in health experiences as well as a lack of cultural and linguistic congruency in care services (e.g. Batista, 2019; Guerin et al. 2018; Martin et al. 2018; Stout et al. 2008).

In Canada, both Anglophone and Francophone older adults living in a linguistic minority context encountered barriers limiting their access to health services (Alimezelli et al. 2013). Despite the fact that Francophone older adults living outside of Quebec were more likely to report poor health in comparison to the older population overall, the vast majority was satisfied with the care they received, although slightly less so than the average Canadian older adult (van Kemenade et al. 2015). Francophone women 65 years and older living in a minority context were just a little bit less satisfied than their male counterparts with regards to the care they received (van Kemenade et al. 2015).

Several studies reported that older Francophones living in a minority context in the province of New-Brunswick had a more limited access to services in terms of housing, transport, leisure, home support and specialized health care or home care (e.g. Dupuis-Blanchard & Villalon, 2013; Dupuis-Blanchard et al. 2014; Simard, 2019). Inadequate service access might further hinder the prospect of ageing in place (e.g. Dupuis-Blanchard et al. 2013), and a lack of public transportation services could fuel the exodus of older people to cities with larger demographic sizes and promote social isolation (Simard, 2019). Further, the predominantly Francophone population living in rural regions of New-Brunswick may be disadvantaged in accessing dementia diagnosis services, which created a risk for underdiagnosis (Pakzad et al. 2013). One study conducted with older adults in the Ontario Home Care System identified both dementia diagnosis and having French as a first language as barriers to receiving physiotherapy services – an association that might be attributed to the high prevalence of French-speaking populations living in rural areas where this service may be more limited (Armstrong et al. 2015). According to another study, almost 75% of Francophones in the province of Manitoba reported not having access to health and social services in French, which may partially result from a lack of French-speaking professionals (de Moissac et al. 2015). Yet another study showed that with significant regional variations, only 38% of Francophone older adults living in a minority context spoke French with their family doctor (van Kemenade et al. 2015). This inability to communicate in one’s first language in health service settings might have left French-speaking older adults more vulnerable as the difficulty of communicating pain and emotions in English was exacerbated by the specialized terminology of medical language (Bouchard et al. 2012). Adequate communication with professionals was thus considered a hallmark of safe practice (Dupuis-Blanchard et al. 2015). Receiving social support from family and community members may have compensated for linguistic gaps in services (Dupuis-Blanchard et al. 2015; Simard et al. 2015).

Batista et al. (2019) found that differences between Ontario nursing homes’ primary language and that of their residents produced negligible outcomes on patients’ health. These outcomes could partially be explained by the characteristics of homes used on behalf of the English-speaking majority and the French-speaking minority, such as the location of the homes and whether they were for-profit organizations or not (Batista et al. 2019). In the province of New-Brunswick, Anglophone establishments tended to document certain parameters of nutritional screening better than Francophone establishments, and vice-versa (Dupuis-Blanchard et al. 2014). Nevertheless, the benefit to Francophone older adults of living in a French environment (Alimezelli et al. 2013; Dupuis-Blanchard et al. 2014; Simard et al. 2015) and their higher utilization of long-term care in certain areas (Guerin et al. 2018) presented compelling evidence for the development of a regulatory framework to ensure the provision of services in the minority language (Forgues et al. 2011; Michaud et al. 2015).

Out of the studies included in this review, one used a cross-country perspective and included both Swedish speakers in Finland and Francophone minorities in Canada. Bilingual consumers found it important to be served in their native language, particularly with regard to high-involvement services, such as medical or financial assistance (Holmqvist & Van Vaerenbergh, 2013). The results showed that even if access to services in one’s native language was especially important in regards to high-involvement services, linguistic minority older adults have been reported to be less willing to switch language even when it comes to low-involvement services, such as being served in stores and cafeterias (Holmqvist & van Vaerenbergh, 2013). A possible explanation was that the lower willingness to switch to a second language could be due to older bilinguals’ reduced ability to maintain fluency in both languages and heightened difficulties with activating their second language (Holmqvist & van Vaerenbergh, 2013).

One of the studies focusing on the Swedish-speaking minority in Finland concluded that experiences of barriers when seeking important health-related information were fairly common, which could lead to the information being experienced as contradictory or confusing (Eriksson-Backa, 2008). The use of difficult medical terms was reported to be the most common barrier among Swedish-speaking older adults when seeking health-related information (Eriksson-Backa, 2008). The second most common barrier was that the information wasn’t provided in one’s own mother tongue or that the language used, for example, in translations, was poor (Eriksson-Backa, 2008).

Among the Welsh studies falling into the societal conditions domain, experiences of language barriers as well as cultural and linguistic congruency were highlighted (Day et al. 2010; 40; Hughes et al. 2009; Martin et al. 2018). Lack of services in one’s native language and communication barriers when accessing services were reported to be strong predictors for decreased well-being, inappropriate care and misunderstandings – especially among older adults with a dementia diagnosis (Martin et al. 2018). Where linguistic and cultural congruity was provided, the patients reported an improvement in the care experience, as well as in their own well-being, due to enhanced communication and cultural understanding (Martin et al. 2018). This further allowed for appropriate care, social stimulation and happiness among Welsh-speaking older adults with a dementia diagnosis (Martin et al. 2018).

Distances between linguistic minority and majority communities as well as communication barriers could lead to experiences of insecurity and a lacking service quality when accessing services (Day et al. 2010; Hughes et al. 2009). According to Hughes et al. (2009), study findings indicated that Welsh-speakers might have no choice but to use English when visiting community pharmacies, since service in Welsh was limited. A majority of Welsh-speakers, however, found it important to speak to a Welsh-speaking pharmacist when worried about a medical condition, since the possibility of speaking Welsh was generally associated with better chances for discussion and experiences of better service (Hughes et al. 2009). One of the included studies addressed differences in place attachment among older adults living in their native communities and those living in retirement areas (Burholt & Naylor, 2005). Historical attachment to community, strong social relations to family and community groups as well as the high use of the Welsh language were reported to generally characterize place attachment among native community residents (Burholt & Naylor, 2005).

## Discussion

Social exclusion is a multidimensional concept and this rapid review identified three main social exclusion domains in the literature: socioeconomic influences, social participation and societal conditions. Exploring existing knowledge along these domains allowed us to see converging as well as diverging patterns in old age social exclusion literature amongst linguistic minorities in Canada, Finland and Wales. Societal conditions including studies on services and health care from a linguistic perspective emerged as the most prevalent category, especially in Canada, whereas studies on social participation were frequent in Finland. Studies conducted in Wales recognized general participation as well as societal conditions. We decided to explore three countries with national linguistic minorities, thus also providing a multiple language perspective on social exclusion. Studying these three contexts made it possible to recognize and examine contextual differences and similarities in the experiences of social exclusion between the different linguistic minority communities. A key question is, however, whether or not it is possible to make comparisons between three different countries with very different linguistic contextual backgrounds – concerning not only the legislative differences but also the variations in the proportion, distribution and vitality of the linguistic minority (see e.g. Baistow, 2011). This review allowed us to draw certain connections between the countries, but also to identify and accentuate some country-specific characteristics concerning social exclusion among linguistic minority older adults. For instance, the high levels of social participation among Swedish-speaking older adults in Finland distinguished them from the other linguistic minorities included in the review (e.g. Nyqvist & Nygård, 2016), whereas in the case of linguistic minorities of Canada, studies focusing on the societal conditions domain were clearly highlighted among the studies included (e.g. Bouchard et al. 2015). Furthermore, the Canadian literature concerning seniors living in a linguistic minority context is unbalanced in favor of Francophones, despite that Anglophone minorities living outside of the Montreal region in Quebec present a health profile that is similar to French-speaking minorities outside of Quebec (Bouchard & Desmeules, 2013). This trend in the literature perhaps reflects the fact that minority Francophones are comparatively more diverse and are spread out over a larger territory than minority Anglophones in Quebec (Alimezelli et al. 2013). Concerning Wales, the sense of community emerged as a significant theme, presenting community cohesion as something of great value to the Welsh-speaking linguistic minority older adults (e.g. Burholt & Naylor, 2005). Furthermore, as in the Canadian context, societal conditions within domains (e.g. healthcare services) were highlighted as a cause of social exclusion in Wales (Day et al. 2010; Hughes et al. 2009; Martin et al. 2018).

Regional variations in levels of social exclusion/inclusion are likely to exist in all three geographical contexts included in this review, with the concentration and vitality of the linguistic group playing a central part. In Wales, the proportion of Welsh-speakers fluctuates significantly between regions, with over 60% of the population speaking Welsh in the county of Gwynedd while less than 10% do so in Monmouthshire (Martin et al. 2018). Similarly, in Finland the Swedish-speaking population cannot be considered to form a homogenous group since they live in very diverse linguistic settings: some of them live in areas where Swedish-speakers constitute a majority, some live in bilingual settings and some live in almost exclusively Finnish-speaking areas (e.g. Kalland and Suominen, 2006). Within the Canadian context, the French-speaking linguistic minority is dispersed over several provinces and territories with significant intra- and inter-provincial/territorial differences in terms of the population’s vitality and geographic concentration, while English-speaking linguistic minorities are concentrated in Quebec with regional differences as well (Bouchard & Desmeules, 2013).

The findings of this review indicate that experiences of social exclusion can be considered fairly common among linguistic minority older adults. Some forms of social exclusion have been studied and reported more extensively among some linguistic minorities than others, making the findings of the review quite homogenous across the three countries. Findings in the societal conditions domain were highly similar across the three countries. Having limited access to social and health care services and health related information in one’s native language was reported to be an issue among all of the included linguistic minority populations. Across the three countries, speaking the minority language was reported to result in limitations in service access, language barriers, longer waiting times as well as receiving flawed/insufficient health information. It is essential to conduct studies with older adults in this respect, since this age group can be considered as especially exposed to a broad range of vulnerabilities (Schröder-Butterfill & Marianti, 2006). Schröder-Butterfill and Marianti (2006) have listed degrading health, lack of physical care and health care, poverty, exclusion from participation in society as well as social isolation and loneliness as factors that might increase the sense of vulnerability. Since this review has drawn parallels between belonging to a linguistic minority and experiencing barriers in service access, socioeconomic inequalities and differences in social participation, linguistic minority status could be perceived as a possible contributor to the before mentioned vulnerabilities.

Our review highlights the plurality in ageing experiences and that language is a social categorization that needs to be taken into consideration. The linguistic minorities included in the review have relatively strong and solid legal rights. In the case of Canada, however, there are regional variations in the degree to which different provinces accommodate the official minority language in health and social services. This is because while the Official Languages Act applies to all federal services, it falls unto provincial governments to regulate the use of official languages in health and social services (Foucher, 2017). Regardless of the rights conferred to the linguistic minorities included in this study, it is shown that they all still experience disadvantages. This could indicate that the policies surrounding the legal rights of the minority populations and the practical implementations of these policies do not go hand in hand. Evidence from the reviewed studies indicate that there is a need for a greater understanding of the minorities’ contextual realities as well as improvements in both the linguistic environments and services in the minority language (e.g. Alimezelli et al. 2013). Furthermore, some authors have called for political/structural reforms in favour of the linguistic minority (e.g. Forgues et al. 2011; Michaud et al. 2015).

We explored three countries with somewhat similar linguistic minority contexts. However, by the end of 2017, there were 55 countries considered bilingual, trilingual or multilingual (Leclerc, n.d.). This implies that there would be a demand for extensive research with a focus on social exclusion among linguistic minority older adults. A potential research interest for future social exclusion/inclusion studies could be to include further countries or communities with bilingual or multilingual ageing populations, such as the autonomous communities of Catalonia and Basque Country in Spain. Broadening the current research to involve other bilingual countries and communities would increase knowledge base for patterns in social exclusion among linguistic communities in order to contribute to a more nuanced picture of ageing societies. Further, future research would benefit from including other linguistic groups such as the indigenous Sami in Finland or the First Nations in Canada as well as other linguistic minorities with a migration background. This could be used to inform potential policy response and to promote social inclusion of diverse and potentially marginalized groups of older people.

### Study Limitations

Although the current rapid review expanded existing knowledge on old age social exclusion from a linguistic perspective, the study does have several limitations which need to be taken into account when interpreting the results.

Since a rapid review methodology was applied, some aspects of a systematic review methodology were omitted or simplified to produce a review within a relatively short time frame. Employing a rapid review methodology allowed for broader inclusion possibilities, but also increased the risk of reporting bias by unintentionally omitting relevant social exclusion studies Additionally, the researchers searched and initially screened the results individually which facilitated the rapid review approach, but also increased the risk of reporting bias. The current review included three countries thus excluding other multilingual contexts. We aimed at including only older adults, however, nine of the included studies included older age groups within a larger age range. Also, in balancing the broad scope of the review, we only included peer-reviewed scientific articles, while excluding the gray literature such as policy briefs, governmental reports as well as book chapters. Concerning Canada, in the Francophone tradition, book chapters are highly valued and account for an important part of scientific production. Furthermore, the omission of reports, such as studies and inquiries commissioned by the Welsh Government and Welsh language Commissioner in Wales and the Finnish Ministry of the Interior and the Finnish Government in Finland may also have limited the number of studies included.

Additionally, studies focusing on professional perspectives (e.g. studies based on samples of social and health care staff working with older adults) on social exclusion among older adults were excluded for this study. However, it should be noted that studies including professional perspectives concerning societal conditions that promote/hinder social inclusion among older adults provide important institutional and organizational perspectives on social exclusion among linguistic minority older adults. Considering not only older adults themselves, but also the institutional and organizational realities that surround them provides a more complete image of the vulnerability that linguistic minority older adults face while navigating through the health care system. Studies which accentuate professional perspectives also endorse interventions, structural and organizational development and policy planning by addressing the challenges professionals face while tending to the needs of linguistic minority older adults.

### Conclusions

The current rapid review has contributed to the literature on old age social exclusion from a linguistic minority perspective. Older people’s social exclusion should be understood from the perspective of diversity and this diversity shapes who is socially excluded and in which ways. Our review brought to light issues of inequality between linguistic majorities and minorities in three different multilingual contexts, highlighting similarities and differences. There is a need for further cross-country comparative research in this area, exploring ways in which older people’s diversity in linguistic background shape the possibility to experience social inclusion.
